# Digital Health Africa (DHA 2025) – Conference Report

**DOI:** 10.3389/fphar.2026.1909596

**Published:** 2026-10-15

**Authors:** Tirhani Maluleke, Catriona Waitt, Veshni Pillay-Fuentes Lorente, Bernd Rosenkranz, Beate Kampmann, Karin Steinecke, Goonaseelan Colin Pillai

**Affiliations:** 1 Roche Products Pty Ltd, Johannesburg, South Africa; 2 Fundisa African Academy of Medicines Development, Cape Town, South Africa; 3 University of Liverpool Department of Pharmacology and Therapeutics, Liverpool, United Kingdom; 4 Makerere University College of Health Sciences, Infectious Diseases Institute, Kampala, Uganda; 5 Division of Clinical Pharmacology, Faculty of Medicine and Health Sciences, Stellenbosch University, Cape Town, South Africa; 6 Institute for Clinical Pharmacology and Toxicology, Charité Universitätsmedizin Berlin, Berlin, Germany; 7 Charité Centre for Global Health, Charité Universitätsmedizin Berlin, Berlin, Germany; 8 Charité Universitätsmedizin Berlin, Corporate Member of Freie Universität Berlin and Humboldt, Universität zu Berlin, Department of Anesthesiology and Intensive Care Medicine, Berlin, Germany; 9 CP+ Associates GmbH, Basel, Switzerland; 10 Division of Clinical Pharmacology, University of Cape Town, Cape Town, South Africa; 11 Pharmacometrics Africa NPC, Cape Town, South Africa

**Keywords:** africa, antimicrobial resistance, artificial intelligence, child health, digital health, health policy, maternal health, pharmacometrics

## Abstract

**Introduction:**

The Digital Health Africa 2025 Conference (DHA-2025, https://digitalhealthafrica.org/) addressed the integration of digital tools within critical global health priorities, including maternal and child health (MCH), antimicrobial resistance artificial intelligence (AI), and pharmacometrics. While the adoption of digital health shows immense potential for improving healthcare access in Africa’s resource-limited environments, its practical application in the public sector remains sub-optimal due to policy misalignment and inadequate systemic investments. Digital health interventions can achieve sustained, large-scale impact when properly supported, as demonstrated by successful platforms like MomConnect in South Africa and MomCare in Kenya. However, successfully scaling these systems beyond initial pilots requires a robust foundation built on three pillars: technical capacity (infrastructure), legal legitimacy (regulation), and effective management (governance). Furthermore, while advanced tools like AI and pharmacometric modeling are now practical realities in healthcare in developed countries, the most significant challenge remains operationalizing and transitioning these sophisticated tools safely from the laboratory to the clinical bedside in Africa.

**Conclusion:**

Scientific advancement in digital health is insufficient on its own; the true test of innovation lies in its safe and practical integration into end-user healthcare delivery. To successfully scale digital health solutions in resource-constrained settings, stakeholders must prioritize cross-continental collaborations, invest heavily in capacity building, and secure long-term commitments to infrastructure and policy development.

## 1 Introduction

The Digital Health Africa 2025 Conference (DHA-2025, https://digitalhealthafrica.org/) presented health technologies, their applications, impact and gaps, and fostered continued collaborations across various health sector fields. While the inaugural Digital Health Africa 2024 Conference (DHA-2024) focused on the impact of digital health, telemedicine, artificial intelligence (AI), and machine learning (Benecke et al., 2024); the main objective of DHA-2025 was to address the integration of digital tools across global health priorities, using maternal and child health (MCH), and antimicrobial resistance (AMR) as tracer areas, alongside system scaling, and advanced methodologies such as AI and pharmacometrics.

As defined by the EU Commission (https://health.ec.europa.eu/ehealth-digital-health-and-care/digital-health-and-care_en) (European Commission, 2026), digital health (also known as eHealth) is the integration of information and communication technologies (ICT) into healthcare to improve disease prevention, diagnosis, treatment, and monitoring. Digital health covers several interconnected domains, such as telehealth and telemedicine (remote consultations), mobile health (mHealth, via smartphone or connected wearable devices), and health informatics (systems that collect, store and manage patient data). Adoption of digital health interventions exhibits the potential for improving access to health services and strengthening the health landscape in the resource-limited environment in Africa. While there may be a number of innovative digital health interventions developed in Africa (see examples in Table 1), the application in the public sector and institutional collaborations remain sub-optimal (Rinke de Wit et al., 2022; Victor et al., 2023). A misalignment of health policies and investment in health (African Institute for Development Policy [AFIDEP], 2023) results in persistent lack of translating research evidence into policies. While the overall investment in health remains low and inadequate, the majority of health investment is allocated to disease management and administrative services. This misalignment necessitates an urgent shift in focus towards the development of systems that can bridge this gap. A critical strategy for addressing this issue involves integrating digital tools, advancing health technology, and fostering meaningful collaborations among stakeholders, as well as a sustainable ecosystem for capacity building in digital health (Kiosia et al., 2024). Digital innovations can streamline healthcare delivery, enhance data management, and enable real-time decision-making, driving more efficient and equitable health outcomes (Abernethy et al., 2022). Investments in health technology, such as telemedicine platforms, AI, and electronic health records, can modernize healthcare systems while making them more accessible. Furthermore, fostering partnerships between governments, private sectors, academia, and community organizations can promote shared expertise and resources, ensuring that policies are both practical and impactful. Aligning health policies with targeted investments is essential to build resilience, improve service delivery, and address the evolving needs of healthcare systems worldwide (Cerf, 2023). Proper implementation of these strategies will ensure resource allocation, reduce disparities, and strengthen overall public health infrastructure.

**TABLE 1:** Digital health interventions in Africa discussed at DHA-2025.

Digital health intervention	Primary purpose	Examples of successful implementation	Limitations & remaining knowledge gaps*	African region
MomCare https://www.pharmaccess.org/momcare/	Links expectant mothers with quality care providers through designated public and private clinics primarily located in remote rural areas or urban slums, facilitating the detection and reduction of pregnancy-related risk at an early stage to prevent further complications	During COVID-19, MomCare added a dedicated set of interventions to the MomCare-bundle within 3 weeks of the outbreak to ensure continued accessibility and good-quality care. This enhanced the ability of providers to reach out to pregnant women at the right time, with the right message and to build internal capacity for improved safety practices during the pandemic	Limitations: The registration and intake processes involved extensive, tedious, and time-consuming paperwork for healthcare providers. A high proportion of participating mothers were illiterate, requiring health workers to manually assist them with forms and signatures Gaps: Because the digital bundle was designed as an overlay to standard clinical services, it is difficult to isolate whether positive maternal outcomes were driven by the platform itself or baseline clinic care (Izudi et al., 2023)	Kenya Tanzania
MomConnect https://www.health.gov.za/momconnect/ (National Department of HealthRepublic of South Africa, 2026)	Aims to support maternal and child health using mobile technology. Through MomConnect, pregnant women and new mothers receive free maternal health messaging via SMS or WhatsApp until their baby is 2 years old and they can use the platform to seek healthcare guidance through an automated chatbot or a human-operated text-based helpdesk	Almost 5 million mothers utilizing public antenatal services have registered on the MomConnect platform in over 95% of public health facilities. In its 10 years of operation, a number of studies have proven MomConnect to positively impact health behaviours, which meaningfully contribute to the Sustainable Development Goals (SDG) of reducing the maternal mortality ratio and ending preventable deaths of newborns and children under 5 years of age (DoH)	Limitations: Mobile numbers used as proxy identifiers prevent longitudinal records; delayed helpdesk response times for indigenous languages Gaps: Long-term impact on clinical maternal mortality; feasibility of transitioning to smartphone app interfaces (Barron et al., 2018; Xiong et al., 2018; Seebregts et al., 2018)	South Africa
BornFyne-Prenatal Management System (PNMS)	An intervention to support maternal health issues in Cameroon, a digital health solution that targets two distinct sets of users: Pregnant women and households defined as user 1 (U1) and healthcare providers and health facilities defined as user 2 (U2)	Potential users of the BornFyne-PNMS platform reported benefits of using such a digital platform to include improved communication, remote consultations, better emergency response network, increased patient engagement and enhanced continuity of care	Limitations: Dependent on smartphone ownership, causing equity gaps; clinic-level electricity and internet deficits; “shopping around” clinic behaviors Gaps: How to maintain financial sustainability and secure national scale in bilingual, decentralized health systems (Obegu et al., 2023; Nkangu et al., 2024)	Cameroon

* Limitations and knowledge gaps were extracted from the literature review.

Several presentations at the Conference demonstrated the usefulness of mathematical modeling to support digital health interventions. A recent virtual international symposium involving experts from multiple disciplines highlighted how this technology is being applied across different scientific fields and can be integrated through collaborative strategies to improve health outcomes (Maluleke et al., 2024). Some of the featured examples that yielded breakthroughs in digital epidemiology included identification of unmapped villages in parts of Nigeria to be vaccinated through evaluation of satellite images as part of the global campaign to eradicate polio; the application of drone technology to determine malaria-infested areas in West Africa; the use of models and simulations to predict patient response to treatment (such as antibiotic resistance patterns), disease outbreaks and spreading patterns.

## 2 Format of the DHA-2025 conference

The success of the hybrid conference, which attracted participants from across the world, was attributed to technology transformation coupled with great collaborative and coordinating expertise. The conference adopted a unique and innovative interactive format, integrating four venues: three physical venues (The Innovation Hub, Pretoria, South Africa; Infectious Diseases Institute (IDI) McKinnell Knowledge Center, Makerere University, Kampala, Uganda; and Charité Center for Global Health​, Charité- Universitätsmedizin Berlin, Germany) plus a virtual platform to accommodate any delegate from across the world. Each venue had designated chairpersons overseeing the overall conduct of the conference at the venue, with sessions facilitated by ‘session chairpersons’. The active presence of digital technical teams, along with a dedicated virtual chairperson, facilitated the seamless operation and coordination of sessions.

DHA-2025 attracted a total of 283 registered delegates during the 2 days as follows.

- 48 delegates registered for the face-to-face attendance with 12 poster presentations at the Innovation Hub, Pretoria, South Africa, representing a diverse range of organizations including academic institutions, research councils, private companies, and healthcare entities.
- 63 delegates attended in person at the Infectious Diseases Institute (IDI) McKinnell Knowledge Center, Makerere University, Kampala, Uganda
- 74 delegates attended in-person at Charité Center for Global Health​, Charité- Universitätsmedizin Berlin, Germany
- 98 delegates live on the virtual platform

The regulatory side-event “Digital Transformation of Medicines Regulation in Africa” (Reyneke et al., 2026) attracted 415 registrations with 336 in attendance, while the satellite Pharmacometrics Africa Conference (https://pmxafrica.org/conference/) had 91 registered delegates. Robust engagement from delegates and audiences across all platforms fostered insightful debates and discussions, which were shared via virtual live chats and open microphone sessions.

## 3 Methodology

The thematic framework of the Digital Health Africa 2025 (DHA-2025) Conference was established on the basis of current global health priorities, with a particular focus on addressing the needs of vulnerable populations. To conceptualize these themes and draw up the comprehensive conference programme, a multidisciplinary scientific committee was formed, comprising clinical and research experts in clinical pharmacology, global health, women’s and children’s health, pediatric infectious diseases and immunology.

Selection and Inclusion Criteria: During the programme design phase, selection criteria were applied to ensure that presented topics focused heavily on recent advancements, technical innovations, and clinical progress in digital health through the thematic lenses of maternal and child health (MCH) and antimicrobial resistance (AMR). While the resulting manuscript does not present a standalone, presentation-by-presentation transcript of every individual speaker, all sessions scheduled in the two-day programme were thoroughly integrated into the synthesis. This includes the opening keynote on global funding, MCH pathways, AMR solutions, real-world scaling, digital foundations, specialized case studies, and advanced artificial intelligence (AI) and pharmacometric methodologies.

The conference report summaries were developed systematically based on these broad thematic groupings. Rather than reviewing presentations in isolation, synthesized overviews highlighted the most critical aspects, systemic challenges, practical enablers, and key outcomes of the discussions that took place during each session.

To establish a robust and representative landscape of digital health interventions (presented in Table 1), inclusion of specific digital tools in this synthesis was based on a systematic framework with the following predefined criteria.

- Direct Programmatic Presentation: The tool was formally presented within the scheduled scientific sessions of the conference (e.g., the BornFyne-PNMS platform in Cameroon, the MomCare in Kenya and Tanzania, the MomConnect tool in South Africa).
- Interactive Session Discussion: The tool was actively highlighted, analyzed, or referenced during panel discussions, roundtables, or participant question-and-answer sessions as an indicative model of scale, integration, or systemic challenge (e.g., MomCare initiative in Kenya).

## 4 Themes and highlights

### 4.1 Operationalizing maternal & child health (MCH) pathways

Despite the immense potential of digital health to improve healthcare access in resource-limited environments, public sector adoption across Africa remains sub-optimal (Rinke de Wit et al., 2022; Victor et al., 2023). This sub-optimality is driven by a misalignment of health policies and low domestic health investments, which frequently prioritizes short-term disease management and administrative services over systematic research-to-policy translation (AFIDEP, 2023). To overcome these policy-to-practice barriers, established digital platforms must be critically evaluated, which represent distinct operational models of scale, ranging from national SMS-based systems and point-of-care clinical standardization tools to highly focused value-based clinic bundles and specialized mobile applications.

The opening session aimed to provide a comprehensive discussion of the current landscape in maternal and child health, specifically highlighting how digital methodologies are being utilized to address identified priorities and gaps. The thematic framing of the day was influenced by the preceding keynote address by Prof. Tulio de Oliveira who addressed the timely concerns around “*Shifting Priorities and the Future of Global Health Funding”.*

The review of MCH realities highlighted the necessity of considering diverse operational environments, underscoring the importance of context-specific challenges and solutions. The discussions reinforced the message that innovation, particularly digital approaches, is central to addressing current MCH gaps. Identified key challenges, learnings and outcomes from MCH discussions included.

- Digital Innovations: Novel tools and digital approaches are urgently needed to bridge current gaps in women and girls’ health
- Systemic Obstacles: A critical hurdle is identifying the underlying systemic obstacles that must be addressed before digital pathways and effective treatments can be universally scaled and prescribed
- Diverse Operational Environments: MCH initiatives must navigate varying conditions, which present unique, context-specific challenges depending on the region
- Standardization and Alignment: Establishing a foundational understanding by clarifying the scope of the MCH field and aligning on key definitions and indicators to ensure measurable outcomes
- Leveraging Institutional Excellence: Relying on established structures that drive MCH progress, such as the mechanisms utilized by The Centre of Excellence in Women and Child Health in Nairobi, Kenya

### 4.2 Infectious disease and AMR: digital tools in the fight for child health

The session examined how digital tools are being integrated into efforts to manage infectious diseases and antimicrobial resistance (AMR), with a distinct focus on applications within pediatric health.

The discussions highlighted the status of AMR and infectious diseases as pressing priorities within child health, demanding specialized digital solutions, as well as the need to move beyond merely acknowledging the problem toward actionable mechanisms that apply digital tools to address these challenges. A recurring theme was that the implementation of technological solutions within health systems remains a significant challenge, given the complexity process of operationalization and embedding new digital health informatics into existing systems. Key challenges, learnings and outcomes from this session included.

- Defining the Burden: a critical lack of comprehensive data to accurately map the crisis of infectious diseases and AMR specifically within the pediatric population
- Operationalization and Translation to Practice: the practical execution of digital solutions is complex, and operationaliz ing and embedding new digital health informatics into existing health systems remains a major obstacle
- Scaling Hurdles: health systems face significant technical, logistical, and systemic hurdles when attempting to scale new digital infrastructure to effectively manage HIV surveillance and infectious diseases
- Evidence-Based Data Acquisition: high-quality data acquisition and interpretation must be prioriti zed to understand current epidemiological and clinical realities, as a prerequisite for developing effective, targeted intervention strategies.
- Actionable Digital Integration: moving beyond acknowledgement of the problem to the integration and application of specialized digital tools to manage urgent pediatric health threats.

### 4.3 Scaling what works–digital health systems in practice (LMIC/HIC)

The reality is that digital interventions, when properly conceived, can achieve substantial reach and sustained impact across varying scales and domains. South Africa’s MomConnect project represents the strength of national, government-backed infrastructure. Operating for over a decade and registered in more than 95% of public health facilities, it has scaled to support over five million mothers through automated text messaging and helpdesks, significantly shifting maternal health-seeking behaviours (Barron et al., 2018; Xiong et al., 2018). Similarly, the success of Rwanda’s RapidSMS project is as a result of government-backed initiatives to achieve national scale, and recorded registration of more than 1.7 million pregnant women, representing 63% of public sector first antenatal care (ANC) visits (Ngabo et al., 2012). However, evidence from local surveys in rural Limpopo indicates that national scale does not guarantee uniform community-level efficacy; grassroots adoption remains fragile and heavily dependent on local health worker dynamics and infrastructural constraints (Muthelo et al., 2023). This highlights the necessity of localized foundational systems (such as Uganda’s electronic maternal and neonatal system, LMNS) to bridge the gap between national platforms and community-level clinics.

The Tanzanian SUSTAIN trial highlighted that the mHealth intervention had almost no impact on changing delivery locations among multiparous women who had previously delivered safely at home, illustrating that standalone digital tools are often insufficient to override deeply established historical clinical behaviors (Hackett et al., 2018).

In contrast, Kenya’s MomCare platform demonstrates the agility of value-based healthcare, linking expectant mothers to specific public and private clinics to manage pregnancy risks early (https://www.pharmaccess.org/momcare/) (PharmAccess Foundation, 2026). MomCare’s strength was highlighted during the COVID-19 pandemic, where a dedicated risk bundle rolled out within 3 weeks successfully sustained skilled deliveries, and reduced complicated births (De Sanctis et al., 2022). Nonetheless, such models require substantial continuous financial backing and remain difficult to replicate in countries lacking a unified, digitally integrated private-public healthcare ecosystem.

Even when technologies are scaled, their real-world impact is heavily constrained by health system capacity, exposing a stark divide between supported pilot environments and national implementation realities. Rwanda’s RapidSMS program demonstrated high reporting compliance and a 27% increase in facility deliveries during its initial pilot phase in Musanze district, which was supported by intensive supervision and error logging (Ngabo et al., 2012). Yet, national evaluations by Musabyimana et al. (2018), showed that RapidSMS had a limited overall impact, with significant positive outcomes restricted solely to districts that received additional international donor (UNICEF) funding and equipment.

In Malawi, the scaling of the Chipatala Cha Pa Foni (CCPF) hotline, which successfully reduced pediatric clinic visits for fever by 15% by promoting home-based management, faced severe institutional resistance (Fotso et al., 2015). The Nursing and Midwives Council of Malawi opposed transitioning registered nurses from the physical bedside to a virtual telephone helpdesk, arguing that it would decay clinical skills and worsen acute clinical staffing shortages at frontline public hospitals.

Meanwhile, Cameroon’s BornFyne-Prenatal Management System (PNMS) offers a dual-user approach that enhances remote consultation and patient engagement (Obegu et al., 2023; Nkangu et al., 2024). However, its implementation highlights a persistent limitation: sophisticated platforms often struggle with the ‘last-mile’ obstacle of adapting complex digital workflows to highly constrained local physical infrastructure and varying human technical capacities. These variations emphasize that digital health cannot be treated as an isolated technological overlay; instead, true scaling requires an ecosystem approach that deeply integrates tools into the broader health environment.

Rather than focusing on patient tracking or financial bundling, the Pregnancy and Newborn Diagnostic Assessment (PANDA) mHealth system evaluated in Ambanja, Madagascar, represents a model focused on point-of-care clinical standardization. By requiring healthcare providers to complete all integrated clinical screening modules before a visit can be closed, PANDA enforces adherence to World Health Organisation guidelines (Benski et al., 2020). This model demonstrated immense strength in improving clinical compliance, achieving near 100% compliance in blood pressure checks and 92.7% in HIV testing, far exceeding Madagascar’s national baselines of 68% and 10% respectively. It also successfully shifted patient behaviour, with second-trimester first antenatal care (ANC) visits rising to 73% compared to the 45% national average.

However, like Cameroon’s BornFyne-Prenatal Management System (PNMS), the PANDA intervention highlights common methodological and knowledge gaps. First, the evaluation of PANDA lacks a randomized control group, relying instead on historical national-level surveys as a reference, which limits the conclusiveness of its findings. Second, the study did not measure long-term clinical health outcomes or mortality, focusing primarily on operational output indicators. Lastly, neither PANDA nor BornFyne has thoroughly explored cost-effectiveness or the long-term durability of these systems compared to traditional paper-based checklists in low-resource settings. These gaps make it difficult to determine whether such localized, hardware-reliant diagnostic ecosystems can achieve long-term financial sustainability and seamless integration into highly constrained national public health grids.

Ultimately, these diverse regional implementations demonstrate that digital health cannot be treated as an isolated technological overlay. True scaling requires an ecosystem approach that deeply integrates tools into the broader health environment, addressing the three co-dependent pillars of technical capacity, legal legitimacy, and effective governance.

Integrating Digital Surveillance to Mitigate Pediatric Infectious Diseases and Antimicrobial Resistance.

The integration of digital health tools to manage pediatric infectious diseases and antimicrobial resistance (AMR) represents a critical frontier in global child health, yet its execution is severely constrained by foundational epidemiological blind spots. Dominant among these constraints is a profound deficit in comprehensive, high-quality pediatric-specific data, which limits the capacity to accurately map the clinical realities of AMR and infectious disease transmission in vulnerable childhood populations. Consequently, systematic data acquisition and empirical interpretation must be prioritized not merely as operational outputs, but as non-negotiable prerequisites for the design of targeted, context-appropriate digital intervention strategies.

Beyond data scarcity, the translation of theoretical technological capabilities into clinical practice faces profound operational barriers. Integrating novel digital health informatics into legacy health systems is an extraordinarily complex process, often hindered by fragmented local infrastructures, rigid clinical workflows, and a lack of interoperability. These translational bottlenecks are further exacerbated by systemic scaling hurdles, as health systems encounter severe technical, logistical, and systemic obstacles when trying to scale digital infrastructure for active HIV surveillance and infectious disease tracking.

To move past the rhetorical acknowledgement of pediatric AMR and infectious disease burdens, global health strategies must shift toward actionable, specialized digital integration. This requires deploying tailored point-of-care tools capable of translating real-time epidemiological intelligence into actionable clinical guidance at the pediatric bedside, thereby bridging the disconnect between high-level policy objectives and frontline pediatric clinical care.

### 4.4 Critically evaluating advanced methodologies (AI and pharmacometrics)

This session explored the enablers that allow digital health to thrive and scale sustainably. Speakers emphasized that successful, widespread digital health deployment depends on a robust, supportive systemic environment spanning technological, legal and educational requirements, including reliable internet access in low-resource settings, secure and interoperable data exchange, and regulatory frameworks that keep pace with implementation. Three co-dependent pillars were identified as central to sustainability: technical capacity (infrastructure), legal legitimacy (regulation), and effective management (governance). Speakers also emphasized the need to share best practices across regions, to avoid the costly duplication of effort.

### 4.5 Case studies and lessons from the field

This session moved from principle to practice, presenting empirical evidence of what works in the field Cameroon’s Born Fyne-Prenatal Management System was featured as a successful example of a digital health intervention that has been rolled out for prenatal care in a specific regional context, while presentations on pediatric and neonatal care demonstrated the extension of digital health into more specialized areas.

- Neonatal monitoring: the successful use of digital tools for monitoring newborns indicated that digital solutions are enhancing surveillance and care for this highly vulnerable population.
- Pharmacological precision: a case study on pediatric dosing/extrapolation using pharmacometrics demonstrated the integration of advanced quantitative methods into digital applications to address the technical and ethical challenges of dosing in children.

The very need for dedicated case studies underscores that translating digital concepts into reliable field practice remains a fundamental challenge, even when implementation succeeds.

### 4.6 Artificial intelligence (AI) in healthcare and research

This session examined current applications of AI in healthcare delivery and medical research. The central message was that AI is no longer a theoretical concept but a practical tool already integrated into health solutions in developed countries and that Africa’s health workforce and technology sector need to build the skills to deploy it. Presentations on how AI tools are applied, monitored and reported in healthcare underlined that AI integration is a cyclical, ongoing process requiring robust governance, continuous monitoring, and transparent reporting of outcomes - not a one-off deployment.

### 4.7 Pharmacometrics, AI and digital tools

This session focused on the use of pharmacometrics to generate reliable dosing recommendations where clinical data are scarce, exploring how quantitative and digital approaches predict drug behavior when ethical and physiological constraints limit standard data collection - using the Dutch Pediatric Formulary as a worked example. Specific modelling applications discussed included.

- Prediction of fetal exposure: novel modelling approaches to predict fetal exposure to drugs, supporting drug safety during pregnancy.
- Risk estimation: pharmacometric models, such as joint longitudinal and time-to-event models, used to estimate specific health risks including preeclampsia risk during pregnancy.
- Physiologically Based Pharmacokinetic (PBPK) modelling: applied in maternal and child health to simulate drug behavior based on physiological processes where clinical data are limited.

The integration of advanced technologies, specifically artificial intelligence (AI) and quantitative pharmacometric modeling, represents a paradigm shift in global pediatric health and maternal medicine. In pediatric and maternal pharmacology, where ethical and physiological constraints severely restrict traditional clinical trials, mathematical modeling serves as an essential tool to bridge critical evidence gaps. This is demonstrated by Physiologically Based Pharmacokinetic (PBPK) modeling and joint longitudinal and time-to-event models, which successfully predict fetal drug exposure and maternal preeclampsia risk. Furthermore, the Dutch Pediatric Formulary highlights how scarce empirical data can be systematically translated into precise pediatric dosing guidelines through quantitative extrapolation.

Yet, while these methodologies are technically mature, a major knowledge and operational gap remains in their translation ‘from model to bedside’. In developed countries, clinical AI has transitioned from laboratory concepts to active service delivery. In Africa, however, the adoption of both AI and sophisticated pharmacometric tools faces a deep disconnect between developers and frontline end-users, such as clinicians and nurses, who often lack the specialized skills, training, and trust required for clinical adoption.

In addition, operationalizing AI in low-resource clinics requires establishing continuous surveillance pipelines to monitor algorithms for real-world accuracy, algorithmic bias, and compliance with clinical safety standards, all of which are hindered by weak regulatory frameworks. To move beyond isolated, pilot-dependent success stories, the digital health foundation must be built upon three co-dependent pillars: technical capacity, legal legitimacy, and effective governance. Without a long-term commitment to infrastructure and policies that can support complex, data-rich clinical decision-support systems, advanced technology will remain confined to the laboratory rather than transforming patient outcomes.

## 5 Challenges and opportunities

Major challenges have been experienced during the practical adoption and implementation of digital health systems as demonstrated by the stark divide between highly supported pilot environments and national implementation realities. In Rwanda, national evaluations of the RapidSMS program showed that positive clinical impacts were strictly restricted to districts receiving additional international donor funding, refresher training, and diagnostic equipment, highlighting that national scaling without intensive localized support fails (van Niekerk et al., 2023). The necessity of systemic support is an indication that scaling is not merely a technical challenge but a governance and infrastructure challenge, where legacy SMS/USSD architectures like South Africa’s MomConnect suffer from severe USSD timeouts during registration and high, non-negotiable commercial texting tariffs that account for approximately three-quarters of ongoing operational costs (Seebregts et al., 2018; Barron et al., 2018). Additionally, using mobile numbers as proxy system identifiers due to USSD timeout constraints fails to support a reliable demographic registry or longitudinal medical record over time (Seebregts et al., 2018). There is also a need for replicable strategies that will address the systemic deficits specific to low-resource environments, moving beyond singular, successful case studies such as the initial pilots of Malawi’s Chipatala Cha Pa Foni (CCPF), which faced severe community skepticism where local populations associated phone-based ‘future-knowing’ delivery predictions with witchcraft (van Niekerk et al., 2023). To facilitate true scaling, initial financial and technical investments needed for piloting successful digital health tools must be followed by long-term commitment to infrastructure and policy development. This is exemplified by Cameroon’s BornFyne-PNMS platform, where the transition to digital systems is constrained by clinic-level electricity deficits, poor internet connectivity, and deep equity gaps, as under-privileged rural households often do not own the smartphones required to access these applications (Obegu et al., 2023). While effective digital tools exist and can achieve localized impact, the complex governance and resource mechanics required to ensure longevity and national reach in low-income settings remains the pre-eminent barrier, as seen in Malawi where the public institutionalization of CCPF stalled due to the lack of official civil service job codes and budgets for nurses operating in a virtual advisory capacity (van Niekerk et al., 2023). To facilitate true scaling, initial financial and technical investments needed for piloting successful digital health tools must be followed by long-term commitment to infrastructure and policy development. While effective digital tools exist and can achieve localized impact, the complex governance and resource mechanics required to ensure longevity and national reach in low-income settings remains the pre-eminent barrier.

The hurdles involving pharmacometrics and pediatric dosing point to the ongoing challenge of integrating highly sophisticated scientific modelling (like predictions for pediatric dosing) into user-friendly digital tools that are safe and effective for healthcare providers in diverse settings. This clinical translational barrier is further compounded by severe human resource constraints and institutional resistance. For example, during the scaling of Malawi’s CCPF hotline, the Nursing and Midwives Council initially opposed the transition of bedside nurses to virtual advisory roles, arguing that virtual patient-provider dialogues carried high clinical risks due to the inability to observe physical signs of illness (van Niekerk et al., 2023). Similarly, in Rwanda, malnutrition tracking via RapidSMS was severely hindered because volunteer community health workers (CHWs) suffered from low motivation and high turnover due to an unpaid task burden, combined with a lack of ongoing training and essential physical equipment like mid-upper arm circumference (MUAC) bands and scales (Musabyimana et al., 2018).

The arrival of AI is recognized as a powerful tool in healthcare. However, skill acquisition, establishment of functional pipelines and governance mechanisms to reconcile the technical requirements of development with the practical needs of clinical end-users remain a major challenge. A critical socio-technical limitation is a profound disconnect between developmental priorities and local behavioral patterns; in Cameroon’s bilingual districts, pregnant women often engage in ‘shopping around’ during antenatal care, bypassing local health facilities to seek care further away due to concerns regarding health worker confidentiality and costs (Obegu et al., 2023).

Beyond the foundational issues around infrastructure and regulation, practical difficulties in integrating digital tools into specific healthcare domains need to be recognized–the integration of digital tools into scientific endeavors. This integration is severely limited by a persistent methodological disconnect in current digital health literature. Evaluations of point-of-care standardization tools, such as Madagascar’s PANDA system, often lack randomized control groups, relying instead on historical national-level surveys, and fail to measure long-term clinical health outcomes (such as maternal and neonatal mortality) or cost-effectiveness compared to traditional paper-based checklists (Benski et al., 2020). Additionally, in cluster-randomized trials like Tanzania’s SUSTAIN, a strong co-linearity exists between smartphone use and increased home-visit frequency, making it methodologically impossible to isolate whether the digital job aid itself or simply the increased physical presence of the community health worker drove improved facility deliveries (Hackett et al., 2018). The digital foundation must therefore be sophisticated enough not only for service delivery, but also for supporting the complex, data-rich requirements of advancing medical research. Lastly, the pre-eminent barrier to translating digital tools into scientific and systemic endeavors is the chronic policy translation failure, where a mere 3.8% of maternal and child health implementation studies successfully influence or improve national government health policies (Till et al., 2023). This underscores the ongoing, complex implementation challenge of enabling specific technical and human factors to successfully embed decision-support systems.

## 6 Recommendations and future considerations

While there are successfully implemented digital health tools, scientific advancement alone is insufficient. The ultimate challenge lies in developing effective governance, policy development, implementation strategies, and communication pathways that allow these complex digital tools and predictive models to be adopted by the end-users (clinicians, nurses, *etc.*) and regulatory bodies to genuinely shape practice. This reinforces that the utility of advanced digital tools is contingent upon their successful, practical, and safe integration into healthcare systems.

Building on the themes above, the following actionable recommendations are proposed for the principal stakeholder groups. Policymakers and regulators should prioritize interoperable, government-owned digital infrastructure over donor-dependent pilots, and establish formal civil-service job codes, budgets and regulatory pathways - including AI-specific governance - for staff working in virtual and digitally-enabled roles. Research funders and implementation scientists should require randomized or quasi-experimental designs, long-term clinical and cost-effectiveness outcomes, and pre-registered evaluation frameworks (e.g., RE-AIM, CFIR, WHO Digital Health Framework) for future digital health studies, to close the evidence gaps identified across the case studies discussed. Clinicians and frontline health workers need structured training and decision-support tools that build trust in AI- and pharmacometric-informed recommendations, co-designed with end-users rather than developed in isolation. Finally, digital health developers should design for infrastructure-constrained settings from the outset - minimizing dependence on continuous connectivity and commercial SMS/USSD tariffs - and build in interoperability with national health information systems to avoid duplicating parallel, unsustainable platforms.

## 7 Conclusion

The pioneering and innovative Digital Health Africa 2025 Conference highlighted the critical importance of integrating technological advancements into capacity building initiatives and emphasized the need for cross-continental collaborations to optimize the resource utilization and fast-track adoption of successfully piloted digital tools in resource-constrained settings. Synthesizing the evidence from mature mHealth implementations across the continent yields several key lessons: while standardized digital tools (such as PANDA in Madagascar and BornFyne-PNMS in Cameroon) improve clinical adherence to healthcare guidelines, and point-of-care job aids (such as Tanzania’s SUSTAIN-CommCare platform) double the odds of skilled facility deliveries among vulnerable primiparous women, their scalability is heavily constrained by high legacy USSD/SMS inventory costs, network timeouts, clinic-level electricity deficits, and community health worker motivation crises under unpaid task burdens.

These systemic barriers highlight major policy implications and institutional bottlenecks. Specifically, digital health integration frequently encounters professional and regulatory resistance, as seen with Malawian nursing councils opposing nurse transition from physical bedsides to virtual hotlines, and public health systems struggling to establish formal civil service job codes and budgets for virtual providers, stalling transition from donor-backed pilots to government-owned registries. To bridge the gap where a mere 3.8% of maternal and child health studies successfully improve national government policies, future implementation priorities must transition from legacy text-based channels to affordable mobile data-driven services that support rich, culturally appropriate audio-visual content. It also highlighted the need for governmental processes and policies to propagate sustainable digital health solutions in Africa.

Various research projects around the theme of the Conference addressing these paradigms are published in the Frontiers in Pharmacology Research Topic “Digital Health in Africa: Advancing Equitable Health for Maternal and Child Health, Rare Diseases, and Infectious Disease Management” (https://www.frontiersin.org/research-topics/73578/digital-health-in-africa-advancing-equitable-care-for-maternal-and-child-health-rare-diseases-and-infectious-disease-management).

## 8 Limitations

Several limitations of this manuscript warrant acknowledgment, primarily arising from its nature as a synthesis of conference proceedings. First, our analysis depends heavily on the presentations delivered during the event, which may lack the exhaustive methodological detail of standard research papers. For example, the emerging discussions on ethics, data privacy, biobanks and AI governance which are very relevant for the implementation of digital healthcare in Africa and elsewhere, were not a major theme of the Conference and therefore not addressed in this publication. Second, the synthesized insights are subject to potential presenter bias and selective reporting, as presentations naturally tend to highlight positive or milestone outcomes. Third, some of the findings discussed remain unpublished in peer-reviewed literature, meaning they have not yet undergone full, independent external evaluation. Finally, given the breadth and concurrent nature of the sessions, it was not possible to critically evaluate all evidence presented during the conference. Consequently, these insights should be interpreted as a thematic mapping of emerging discussions rather than a definitive systematic review.
